# Quantification of the healthy worker effect: a nationwide cohort study among electricians in Denmark

**DOI:** 10.1186/1471-2458-11-571

**Published:** 2011-07-18

**Authors:** Lau C Thygesen, Ulla A Hvidtfeldt, Sigurd Mikkelsen, Henrik Brønnum-Hansen

**Affiliations:** 1 National Institute of Public Health, University of Southern Denmark, Copenhagen, Denmark; 2 Department of Public Health, Social Medicine Section, University of Copenhagen, Copenhagen, Denmark; 3 Department of Occupational and Environmental Medicine, Copenhagen University Hospital, Bispebjerg, Copenhagen, Denmark

## Abstract

**Background:**

The healthy worker effect (HWE) is a well-known phenomenon. In this study we used the extensive registration of all Danish citizens to describe the magnitude of HWE among all Danish electricians and evaluated strategies for minimizing HWE bias of the association between occupation and mortality.

**Methods:**

All Danish male citizens aged 26-56 years in the period 1984-1992 were followed for three years in several registers. We evaluated HWE bias among electricians because they were unexposed to detrimental occupational exposures. We compared electricians to three reference groups (general population, construction industry and carpenters/brick layers) and utilized analytical methods for minimizing HWE bias (lag time analyses, age-stratified analyses, marginal structural model and restriction to employed, newly employed or long-term workers).

**Results:**

The mortality rate was higher among electricians, who the year following active employment received incapacity benefits or were on long-term sick leave. Electricians receiving incapacity benefits, on long-term sick leave, unemployed, or with increased comorbidity index had lower odds of re-employment. Electricians had lower mortality rate (rate ratio,0.60;95%CI,0.52-0.69) compared to the general population, while electricians leaving employment had increased mortality (1.90;1.50-2.40). Adjusting for several social events slightly attenuated the estimates, while the marginal structural model did not minimize bias. Electricians had the same mortality as the construction industry and carpenters/brick layers. Mortality was comparable to the general population after three or more years of lag time.

**Conclusions:**

In this nationwide study, employment as electricians had marked effect on mortality. Appropriate reference selection and lag time analyses minimized the HWE bias.

## Background

Occupational studies of employees under specific conditions may be used to describe e.g. how certain physical or chemical exposures influence morbidity and mortality. Since 1885 [1] it has been recognized that persons employed have a lower morbidity and mortality compared to the general population, because relatively healthy individuals are likely to gain employment and to remain employed, while severely ill and chronically disabled are ordinarily excluded from employment [2,3]. This phenomenon has been termed the *healthy worker effect (HWE) *or *healthy worker survivor bias *[4]. Several occupational studies have shown a negative association between exposure and mortality [5-9], mostly pronounced for risk factors closely correlated with length of occupation or when length of employment was used as exposure proxy [8].

HWE has previously been reported to be strongest for young age groups and at the beginning of employment [5]. It has also been suggested that HWE bias is strongest for diseases of the cardiovascular, respiratory, digestive and urinary systems and weak for malignant diseases [5,10,11]. Analytical suggestions to minimize HWE bias have been suggested, e.g. not to use the general population as reference group [12], to adjust for covariates associated with employment status (e.g. incapacity or sick leave benefits) [13], restricting the analysis to active employees [12,14] or introducing a lag time between employment and mortality [13,15].

We often wish to compare persons with high exposure (e.g. persons exposed for several years) with persons with no or low exposure, e.g. persons in the reference population or persons in the same occupation but with lower exposure. In other words, we wish to estimate the influence of the cumulative exposure in one analysis.

In this study we follow a nationwide cohort of electricians for three years to evaluate the influence of employment in year 0 and 2 on all-cause mortality in year 3, while taking account of covariates in year 1. We study these effects among electricians because, at least to our knowledge, electricians are a trade without any generally acknowledged occupational diseases (which may seriously affect their health). We would therefore expect similar mortality among electricians as selected reference groups if the bias caused by HWE (HWE bias) is minimized. We use the extensive registration of all Danish citizens in registers to describe the magnitude of HWE by estimating the influence of termination of employment on subsequent mortality and the probability of re-employment among persons with terminated employments (selection out of and into the working force). We utilize analytical methods to minimize HWE bias offered by a number of authors [3,5,8,9,12-15], which will be further explained below.

## Methods

**Figure 1:**
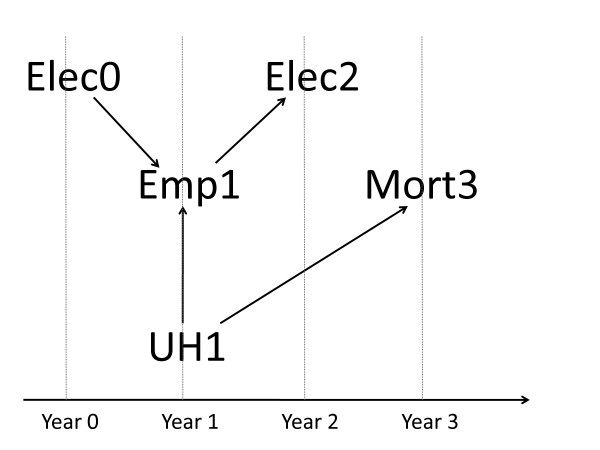
**Proposed structure of employment as electrician in year 0 (Elec0) and year 2 (Elec2), underlying health in year 1 (UH1), employment in year 1 (Emp1) and outcome (mortality) in year 3 (Mort3)**. *Text below figure: *Employment as electrician in year 0 (Elec0) increases the probability of employment in year 1 (Emp1), which again increases the probability of employment as electrician in year 2 (Elec2). Underlying health (UH1) is associated with employment (Emp1) and outcome (mort3).

In this study we follow a nationwide cohort of electricians for three years (baseline in year 0 with follow-up until year 3) to evaluate the influence of employment in year 0 and 2 (proxy measure of cumulative exposure) on the outcome in year 3 (all-cause mortality), while taking account of covariates in year 1 (see Figure 1 for the basic setup). By this setup the influence of the simplest cumulative employment measured in only two years (Elec0 in year 0 and Elec2 in year 2) was evaluated with respect to mortality in year 3 (Mort3) and adjusted for potential confounders in year 1. This presentation makes interpretation of the cumulative employment simple and the results are therefore easier to interpret.

We use employment as electricians in year 0 and 2 (Elec0 and Elec2) as a proxy measure of exposure, which, to make things simple, is assumed to be unrelated with outcome (here mortality in year 3: Mort3), shown by no arrow from Elec0 and Elec2 to Mort3 in Figure 1. Underlying health in year 1 (UH1), which is rarely measured, is associated with mortality at some later time (Mort3) and with the person's employment status in any occupation in year 1 (Emp1) [14,16], which again is associated with future employment as electrician (Elec2), because you are less likely to be employed as electrician in year 2 if not employed in year 1. Furthermore, Emp1 is partly determined by former employment as electrician (Elec0) [6,15], because employees in year 0 have a higher probability of being employed in year 1. When a confounding factor (such as employment) determines subsequent employment and is determined by previous employment, then standard analyses which estimate disease incidence as a function of cumulative employment may not validly estimate the true effect of employment, even when adjustment is made for the confounder [6,15,16].

We included all Danish male citizens aged 26-56 years on January 1^st ^from 1984 to 1992, who survived until January 1^st ^next year. The same person could therefore contribute during several years. We only included males, since only 200 women were electricians during this period compared to 29,613 men in the same period. Each Danish citizen has a unique personal identification number, which makes linkage on individual level between several registers possible.

We obtained information on employment from the Integrated Databank on Research in the Labor Market [17], which includes all employed persons and their place of employment in Denmark with annual information since 1980. Persons employed could be described both by information on the specific employment (occupational position code for most important employment during a calendar year), highest vocational education by 1 January and information on the place of employment (line of business by the end of November). By this register we defined electricians as persons employed as skilled electronics mechanic with the highest educational level as skilled electrician or electronics mechanic. Table 1 includes the relevant codes.

**Table 1:** Definitions of persons employed as electricians, in the construction industry and as carpenters/brick layers

	Electricians	Construction industry	Carpenters and brick layers
Occupational position code (the variable NyStGr at Statistics Denmark [17])	4585X	4403X 45810-45860 4587X 4593X 4595X 4673X 46810-46859 4689X 46930-46959 *Excluding:* 4583X 4683X 4694X	4595X
Highest vocational education (the variable ekfsp at Statistics Denmark [17])	35536520 35536530 35536540 35547040 35548030	*Excluding:* 6559XXXX (master of engineering) 50590010-50594510 (bachelor of engineering) 50595010 (building technician)	35534010 35534053 35534015 35534040 35544060 35533520 35544050 35544070 35531510
Place of employment (line of business) (the variable branche1 at Statistics Denmark [17])	45XXXX Missing value	200000-220000 26XXXX 45XXXX Missing value	45XXXX Missing value

We included three comparison groups (reference groups): (1) The *general Danish male population *in the same age-span as specified above, (2) the *construction industry *defined as persons employed in the building and construction sector or the timber, paper, stone, clay or glass industries excluding bachelors or masters of engineering and building technicians, and (3) *carpenters *and *brick layers *defined as persons employed as carpenter or brick layer with highest educational level as skilled carpenter or brick layer (Table 1 for codes). The comparison groups were selected as groups with whom electricians share certain work related and socioeconomic characteristics. The three comparison groups reflect increasingly homogeneous groups with increasing similarity to electricians.

We followed each Danish citizen in Statistics on Social Benefits [18], which contains monthly information on all Danish citizens receiving any income substitution since 1984. We included information on incapacity benefits, which was transferred to persons with at least 50% permanently reduced ability to work [19]. We included information on sick leave, i.e. information on the duration of sickness reimbursements from the municipality during a specific year [20]. Since 1973 the Danish municipalities have had the financial responsibility of sickness benefits, but with 75% reimbursements from the state in the period studied here. The sickness absence benefits cover all citizens. The benefits are paid by the employer during a specified period (employer period). After this period the benefit paid by the employer is reimbursed by the municipality. Qualifying days, benefits and reimbursement level and duration have changed several times since 1973 and have been somewhat different for white and blue collar worker and for private and public sector employees [20]. We categorized this variable as 0-13 days, 14-91 days (short-term sick leave) and more than 91 days (long-term sick leave) during the same year.

Furthermore, we included information on unemployment measured as number of weeks unemployed divided by number of weeks during the year resulting in a number between 0% and 100%. Short and long term unemployment was characterized as 21-80% or more than 80% and not having received sickness benefits more than 13 days during that specific year.

In the following we use the term social event for persons who received incapacity benefits, were on sick leave or were unemployed. A person was considered employed in a specific year if he did not receive incapacity benefits, was employed at least 20% of the year and had less than 91 days of sick leave.

Information on comorbidity was given by the Danish National Patient Register, which is a register of all inpatient hospitalizations in Denmark since 1977 [21]. For each person the Charlson's Comorbidity Index, modified for the International Classification of Diseases, eighth revision, was used to define comorbidity [22]. To estimate the comorbidity in a given year, we used discharge diagnoses during the previous two years.

Information on mortality, emigration and disappearance were obtained from the Danish Civil Registration System [23].

When using the general population as a reference group, we compared persons employed as electricians in year 0 *and *year 2 (Elec0 and Elec2) and persons employed as electricians in year 0 *or *2 with persons not employed as electricians. The first group had 'high exposure' (electrician both in year 0 and 2). The second group had 'low exposure' (electrician only in year 0 or year 2), which was divided into persons only employed as electricians in year 0 and persons only employed as electricians in year 2. The last group was 'not exposed' (not employed as electrician). When using the construction industry and carpenters/brick layers as reference groups, we made pair wise comparisons between electricians and reference group with the same employment, e.g. persons employed as electricians only in year 0 were compared with construction industry workers only employed in year 0.

We performed two groups of analyses: Firstly, we described HWE by estimating the mortality rates among electricians in year 0 and year 1 compared to electricians in year 0, who were not employed as electricians in year 1, e.g. persons unemployed or persons receiving incapacity benefits. Furthermore, we estimated the odds ratio of returning to work as electrician in year 2 among electricians in year 0 after experiencing any social event or having increased comorbidity index in year 1.

Secondly, we utilized analytical suggestions to minimize HWE bias of the association between employment as electrician and mortality. We used three different reference groups [12], adjusted for social events (incapacity benefits, sick leave and unemployment), employment status, age, and calendar year [13] and restricted the analysis to active employees [12,14,24]. We stratified the analyses by age (< 40, 40-49 and 50+ years) [5,11]. We carried through latency analyses [13,15,24], where we lagged the influence of employment on the mortality rate by 1, 3, 5 and 7 years [13,25]. We also restricted the analysis to newly employed electricians excluding workers employed the year before study inclusion [5,9] and to long-term electricians employed as electricians for at least two out of the preceding three years before study inclusion [5].

Since employment in year 1 (Emp1) may both determine subsequent employment as electrician (Elec2) and be determined by previous employment as electrician (Elec0) (Figure 1), standard regression analyses may not validly estimate the cumulative employment effect [6,15,16]. One analytical solution may be marginal structural models (MSM), which can estimate the effect of employment in the presence of time-dependent covariates that may be simultaneously confounders and intermediate variables [26,27]. This was done by an age- and calendar year adjusted and a weighted Poisson regression model in which the weights were estimated as the inverse probability of being employed as electrician in year 2. Briefly, a logistic regression of being electrician in year 2 was regressed on social events, employment status and Charlson's Comorbidity Index (covariates) in year 1 and used to obtain the predicted probability of being electrician. The weights were calculated as the inverse probability of being electrician, where age and calendar year were added in the numerator and denominator to stabilize the weights. Assuming no unmeasured confounders, this weighting procedure created a pseudo-population where being electrician was unconfounded by the covariates mentioned above [26]. Therefore the weighted Poisson regression model was only adjusted for age and calendar year.

Logistic regression was used to model the odds ratio of being employed as an electrician. Poisson regression was used to model mortality during follow-up, with logarithmic transformation of person-years of risk as offset value. For these analyses, we used the PROC GENMOD procedure of SAS version 9.1 (SAS Institute Inc.; Cary, NC). Mortality rate ratios (MRR), odds ratios (OR) and 95% confidence intervals (95%CI) were calculated.

## Results

**Figure 2:**
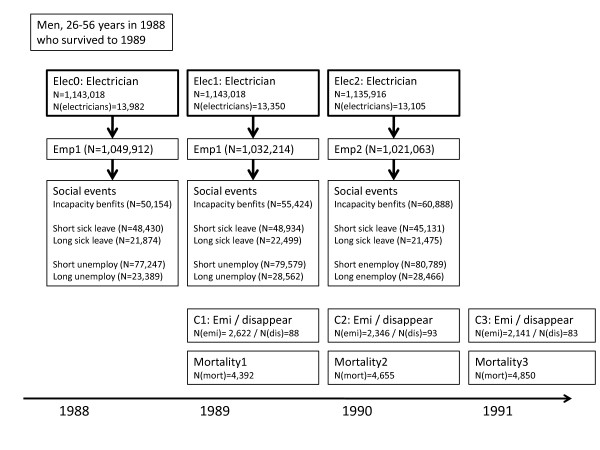
**Employment in general and as an electrician, social events and mortality, men aged 26-56 in 1988 who survived to 1 January 1989**.

To present the data, we provide the numbers for one year (1988). The number of men on January 1^st ^1988 who survived until January 1^st ^1989 was 1,143,018 including 13,982 electricians (Figure 2). The number of employed persons decreased from 1,049,912 in 1988 to 1,021,063 in 1990, while the number of persons receiving incapacity benefits increased during the same period. The number of electricians decreased to 13,105 in 1990. The number of emigrated persons decreased during the period, while the number of deaths increased. These trends probably reflected an aging population during the three years of follow-up.

Among electricians at baseline (year 0) not employed as electricians in year 1, the mortality rate in year 2 was significantly higher than persons employed as electricians both in year 0 and 1 (MRR = 4.04; 95%CI, 2.88-5.66) and very high for persons on long-term sick leave (15.7; 11.0-22.4) and persons receiving incapacity benefits (12.4; 7.18-21.4) (Table 2). Unemployed persons also had increased mortality. The markedly increased mortality among persons on sick leave and receiving incapacity benefits was observed for all follow-up periods (up to six years), while the MRR decreased for later years for persons employed not as electricians.

**Table 2:** Electricians in year 1984-1992 (year 0), their employment category the next year (year 1) and the mortality rate ratio in year 2, 3-4 and 5-6 years, Denmark, men

Employment category year 1	Mortality year 2	Mortality year 3-4	Mortality year 5-6
	MRR (95%CI)	MRR (95%CI)	MRR (95%CI)
Employed as electrician	1 (ref)	1 (ref)	1 (ref)
Employed not as electrician	4.04 (2.88-5.66)	2.14 (1.69-2.72)	1.34 (1.03-1.73)
Short-term sick leave	3.95 (2.62-5.98)	4.07 (3.10-5.34)	4.25 (3.23-5.61)
Long-term sick leave	15.7 (11.0-22.4)	13.2 (10.3-16.9)	13.0 (10.1-16.9)
Short-term unemployment	1.99 (1.26-3.14)	1.92 (1.42-2.62)	1.80 (1.31-2.48)
Long-term unemployment	1.91 (0.89-4.11)	2.51 (1.69-3.74)	2.69 (1.85-3.92)
Incapacity benefits	12.4 (7.18-21.4)	10.2 (7.62-13.6)	11.7 (9.05-15.0)
			
Number died	252	550	526
Number emigrated	54	112	90
Number disappeared	0	3	2

MRR, mortality rate ratio; 95%CI, 95% confidence interval. All estimates are adjusted for age (5-year groups) and calendar year. Each person-interval could be in several categories at year 1. Therefore all MRRs are pairwise comparisons between the respective category in year 1 compared to electricians in year 1 (reference). The number in the reference group differed for each pairwise comparison.

Electricians in year 0, who were on long-term sick leave in year 1 had lowered OR of being employed as electrician in year 2 compared to persons employed as electrician (OR = 0.11; 0.10-0.12) (Table 3). Persons receiving incapacity benefits had even lower OR. Persons not employed as electricians in year 1 had decreased odds of being employed as electricians in year 2 (OR = 0.02). Persons with high comorbidity index (index = 3+) in year 1 had lower OR of being employed as electricians in year 2 (0.16; 0.11-0.23) compared to persons with no comorbidity (index = 0). Lower OR was also observed for comorbidity index 1-2.

**Table 3:** Electricians in year 0, who survived until year 3, their employment category the next year (year 1) and their odds of being electricians at year 2, Denmark, men

Employment status in year 1 among electricians in year 0	OR of being an electrician at year 2
	OR (95%CI)
Employed as electrician	1 (ref)
Employed not as electrician	0.02 (0.02-0.02)
Short-term sick leave	0.50 (0.45-0.54)
Long-term sick leave	0.11 (0.10-0.12)
Short-term unemployment	0.29 (0.27-0.31)
Long-term unemployment	0.26 (0.22-0.29)
Incapacity benefits	0.02 (0.01-0.02)
Comorbidity index 1-2 (medium)	0.29 (0.26-0.32)
Comorbidity index 3+ (high)	0.16 (0.11-0.23)

OR, odds ratio; 95%CI, 95% confidence interval. All estimates are adjusted for age (5-year groups) and calendar year. Each person-interval could be in several categories at year 1. Therefore all ORs are pairwise comparisons between the respective category compared persons employed as electricians in year 1 (reference). The number in the reference group differed for each pairwise comparison.

**Figure 3:**
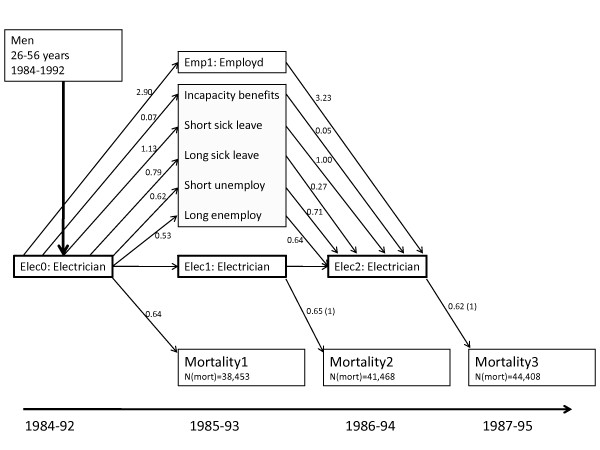
**Mortality, social events and employment for electricians aged 26-56 years in 1984-1992 compared to the general population**. *Text below figure: *Numbers at arrows are odds ratios estimated by logistic regression adjusted for age (5-year groups), calendar year and preceding variables. (1) Only adjusted for age (5-year groups) and calendar year.

The tracking of being an electrician in year 0 (1984-92), to social events in year 1 (1985-93) and back to being an electrician in year 2 (1986-94) is shown in Figure 3. When comparing electricians with the general population, electricians in year 0 had increased odds (OR = 2.90) of being employed in year 1 and being on short-term sick leave (OR = 1.13), while decreased odds of receiving incapacity benefits, being unemployed or on long-term sick leave (Figure 3). Persons receiving incapacity benefits (OR = 0.05), on long-term sick leave (OR = 0.27) and unemployed had lower odds of being electricians in year 2. In all three years, persons employed as electricians had decreased mortality rate compared to the general population (e.g. in year 1 MRR = 0.65).

The mortality rates (MR) were lowest for electricians in year 2 and highest for persons who were electricians only in year 0 (Table 4). In Model 1 with adjustment for age and calendar year, an increased mortality rate was observed for electricians only year 0 (MRR = 1.90; 1.50-2.40) and decreased MRR for electricians both in year 0 and 2 (MRR = 0.60; 0.52-0.69) compared to the general population (not electricians).

**Table 4:** Electricians versus the general population, Denmark, mortality rate 1987-1995, men

Exposure	#Death	RT	MR	Model 1	Model 2	Model 3	Model 4
				MRR (95%CI)	MRR (95%CI)	MRR (95%CI)	MRR (95%CI)
Not electrician	44,117	10,033,289.7	439.7	1 (ref)	1 (ref)	1 (ref)	1 (ref)
Electrician only year 0	70	13,394.7	522.6	1.90 (1.50-2.40)	1.80 (1.42-2.27)	2.17 (1.62-2.91)	1.91 (1.51-2.41)
Electrician only year 2	25	9,649.5	259.1	0.90 (0.61-1.34)	1.12 (0.75-1.65)	1.08 (0.67-1.73)	2.03 (1.59-2.59)
Electrician year 0 and 2	196	106,846.9	183.4	0.60 (0.52-0.69)	0.77 (0.67-0.89)	0.80 (0.68-0.94)	0.67 (0.59-0.77)

RT, risk time - person years of risk; MR, mortality rate per 100,000 person-years; MRR, mortality rate ratio; 95%CI, 95% confidence interval.

Model 1: Adjusted for age (5-year groups) and calendar year.

Model 2: Adjusted for employment status, incapacity benefits, sick leave and unemployment in year 1, age (5-year groups) and calendar year.

Model 3: Only persons with employment status = 1 in year 2 were included. Adjusted for employment status, incapacity benefits, sick leave and unemployment in year 1, age (5-year groups) and calendar year.

Model 4: Marginal structural model with weights estimated as described in the Methods section. Adjusted for age (5-year groups) and calendar year.

In the multivariable adjusted model (Model 2) an attenuation of risk estimates was observed, but significantly decreased MRR for electricians in year 0 and 2 and increased MRR for electricians only in year 0 was still observed. In Model 3, only persons employed in year 2 were included, showing stronger risk estimates for electricians only in year 0 (MRR = 2.17; 1.62-2.91) and electricians in year 0 and 2 still had significantly decreased mortality rate (0.80; 0.68-0.94) (Table 4). In Model 4, MSM was implemented with estimated weights of 1.00, 0.78 and 76.43 as the mean, minimum and maximum weights. The weighted Poisson model showed increased risk for electricians either in year 0 or in year 2 and decreased risk for electricians both in year 0 and 2.

The mortality rate among persons employed in the construction industry was slightly lower (MR = 408.5 per 100,000 person-years (10^5 ^p-yrs) - Table 5) compared to the general population (439.7/10^5 ^p-yrs - Table 4). Electricians had decreased MRR compared to the results with the general population as reference group, e.g. in Model 2 the relative risk was equal for electricians compared to persons employed in the construction industry (MRR = 1.01; 0.87-1.19). Increased risk was observed both for electricians only in year 0 and electricians both in year 0 and 2 in the MSM model (Table 5 - model 4).

**Table 5:** Electricians versus the construction industry, Denmark, mortality rate 1987-1995, men

Exposure	#Death	RT	MR	Model 1	Model 2	Model 3	Model 4
				MRR (95%CI)	MRR (95%CI)	MRR (95%CI)	MRR (95%CI)
Not electrician	2,571	629,336.2	408.5	1 (ref)	1 (ref)	1 (ref)	1 (ref)
Electrician only year 0	70	13,394.7	522.6	1.13 (0.89-1.45)	1.29 (1.00-1.65)	1.39 (1.01-1.90)	1.40 (1.12-1.75)
Electrician only year 2	25	9,649.5	259.1	0.79 (0.53-1.19)	0.84 (0.56-1.27)	0.74 (0.45-1.21)	0.82 (0.55-1.21)
Electrician year 0 and 2	196	106,846.9	183.4	0.90 (0.77-1.05)	1.01 (0.87-1.19)	0.95 (0.80-1.14)	1.18 (1.03-1.36)

RT, risk time - person years of risk; MR, mortality rate per 100,000 person-years; MRR, mortality rate ratio; 95%CI, 95% confidence interval.

Model 1: Adjusted for age (5-year groups) and calendar year.

Model 2: Adjusted for employment status, incapacity benefits, sick leave and unemployment in year 1, age (5-year groups) and calendar year.

Model 3: Only persons with employment status = 1 in year 2 were included. Adjusted for employment status, incapacity benefits, sick leave and unemployment in year 1, age (5-year groups) and calendar year.

Model 4: Marginal structural model with weights estimated as described in the Methods section. Adjusted for age (5-year groups) and calendar year.

The mortality rate among carpenters and brick layers was 301.3/10^5 ^p-yrs (Table 6). In general the results were the same as with the construction industry as reference group except for newly employed electricians (electricians only in year 0), where electricians had a markedly lower mortality rate compared to newly employed carpenters and brick layers (reference group). Adjustment for all social variables, restricting the analysis to employed persons and implementing MSM did not change these results.

**Table 6:** Electricians versus carpenters and brick layers, Denmark, mortality rate 1987-1995, men

Exposure	#Death	RT	MR	Model 1	Model 2	Model 3	Model 4
				MRR (95%CI)	MRR (95%CI)	MRR (95%CI)	MRR (95%CI)
Not electrician	738	244,977.7	301.3	1 (ref)	1 (ref)	1 (ref)	1 (ref)
Electrician only year 0	70	13,394.7	522.6	0.99 (0.75-1.30)	1.15 (0.87-1.53)	1.39 (0.95-2.04)	1.20 (0.92-1.57)
Electrician only year 2	25	9,649.5	259.1	0.50 (0.32-0.77)	0.52 (0.33-0.81)	0.46 (0.27-0.79)	0.49 (0.32-0.76)
Electrician year 0 and 2	196	106,846.9	183.4	1.06 (0.89-1.25)	1.24 (1.04-1.48)	1.17 (0.96-1.43)	1.30 (1.11-1.53)

RT, risk time - person years of risk; MR, mortality rate per 100,000 person-years; MRR, mortality rate ratio; 95%CI, 95% confidence interval.

Model 1: Adjusted for age (5-year groups) and calendar year.

Model 2: Adjusted for employment status, incapacity benefits, sick leave and unemployment in year 1, age (5-year groups) and calendar year.

Model 3: Only persons with employment status = 1 in year 2 were included. Adjusted for employment status, incapacity benefits, sick leave and unemployment in year 1, age (5-year groups) and calendar year.

Model 4: Marginal structural model with weights estimated as described in the Methods section. Adjusted for age (5-year groups) and calendar year.

The decreased mortality rate among electricians in year 0 and 2 compared to the general population was largest among the youngest age group 29-39 years, MRR = 0.61 (0.46-0.80) and approached unity for the oldest age group 50-59 years, MRR = 1.00 (0.81-1.23) (Table 7). The significantly increased mortality rate among persons only employed as electricians in year 0 was only observed for the oldest age group (MRR = 2.74; 1.99-3.79).

**Table 7:** Electricians versus the general population, Denmark, men: stratified by age

Exposure	Age 29-39	Age 40-49	Age 50-59
	MRR (95%CI)	MRR (95%CI)	MRR (95%CI)
Not electrician	1 (ref)	1 (ref)	1 (ref)
Electrician only year 0	1.38 (0.87-2.19)	1.33 (0.80-2.20)	2.74 (1.99-3.79)
Electrician only year 2	0.92 (0.44-1.93)	1.12 (0.56-2.24)	1.45 (0.78-2.70)
Electrician year 0 and 2	0.61 (0.46-0.80)	0.78 (0.60-1.01)	1.00 (0.81-1.23)
Number died	6,772	11,902	25,734

MRR, mortality rate ratio; 95%CI, 95% confidence interval. Adjusted for employment status, incapacity benefits, sick leave and unemployment in year 1, age (5-year groups) and calendar year.

When inferring lag time the mortality rates among electricians were comparable to the general population three and more years after exposure, e.g. MRR among electricians in year 0 and 2 for three years of lag time was 0.92 (0.82-1.04) (Table 8). The increased mortality rate among electricians only employed in year 0 was also insignificant after three years.

**Table 8:** Electricians versus the general population, Denmark, men: latency analyses

Exposure	Latency 0 years	Latency 1 years	Latency 3 years	Latency 5 years	Latency 7 years
	MRR (95%CI)	MRR (95%CI)	MRR (95%CI)	MRR (95%CI)	MRR (95%CI)
Not electrician	1 (ref)	1 (ref)	1 (ref)	1 (ref)	1 (ref)
Electrician only year 0	1.80 (1.42-2.27)	1.56 (1.21-2.00)	1.29 (0.98-1.71)	0.81 (0.55-1.20)	0.83 (0.51-1.33)
Electrician only year 2	1.12 (0.75-1.65)	1.19 (0.82-1.74)	1.03 (0.66-1.59)	0.87 (0.52-1.48)	0.82 (0.44-1.53)
Electrician year 0 and 2	0.77 (0.67-0.89)	0.83 (0.73-0.95)	0.92 (0.82-1.04)	0.94 (0.84-1.05)	0.93 (0.84-1.03)
Number died	44,408	47,717	54,345	61,526	69,369

MRR, mortality rate ratio; 95%CI, 95% confidence interval. Adjusted for employment status, incapacity benefits, sick leave and unemployment in year 1, age (5-year groups) and calendar year.

When restricting the analysis to newly employed electricians, the mortality rate ratios were similar to the general population after adjusting for the social events (not shown). The number of outcomes was small in this analysis though. When restricting the analysis to long-term electricians, the MRR showed increased risk for electricians only in year 0 (1.90; 1.49-2.42) and decreased MRR for electricians both in year 0 and 2 (0.79; 0.68-0.91). These results were similar to the results in Table 4.

## Discussion

In this study we characterized HWE in a nation-wide cohort of electricians as the selection out of and into the working force and evaluated different methods for minimizing the influence of HWE bias. There was a strong selection out of the cohort in that increased mortality among electricians leaving the working force was observed especially among persons on long-term sick leave and persons receiving incapacity benefits. The odds ratio of re-entering the electrician work force was low for persons on long-term sick leave, unemployed, receiving incapacity benefits and with increased comorbidity index. A decreased mortality rate was observed among electricians compared to the general population. Adjustment for several social variables and comorbidity index slightly attenuated this difference. When including relevant reference groups (construction industry and carpenters/brick layers) the differences attenuated although electricians who left employment had increased mortality compared to workers in the construction industry and electricians who entered employment had lowered mortality compared to carpenters/brick layers. Electricians had the same mortality rate as the general population when inferring lag time of three or more years. Our findings that appropriate reference group selection and lag time analyses may minimize HWE bias should be considered in other occupational studies.

Other occupational cohort studies have shown lower mortality among persons employed compared to the general population. The standardized mortality ratio was often reported as approximately 80 [5], from 30 to 50 [8] or under 100 [14] for all-cause mortality during active employment and rising to 240 the year following active employment [8]. These estimates of decreased mortality during active employment seem consistent with our findings of MRR = 0.60 for the age and period adjusted model. We also observed increased mortality the year after employment termination varying from MRR = 1.99 for electricians experiencing short-term unemployment to MRR = 15.7 for electricians on long-term sick leave. We observed that employment as electrician was protective for the youngest age-group and employment termination was detrimental for the oldest. In line with this, Flanders showed that time-since-hire was associated with decline in health and could therefore cause an artificial adverse effect of cumulative exposure [28] in the opposite direction as HWE. Consistently, Howe argues that the maximal lowered mortality is observed during the first five years of employment [10]. These results seem in line with ours indicating an effect modification by age.

We evaluated different methods for minimizing the influence of HWE bias and found that selection of an appropriate reference group and latency analyses were effective in minimizing the lower mortality among electricians and the increased mortality among electricians who stopped being electricians. Selection of an appropriate reference group has been suggested elsewhere [5,12] assuming that workers in other rather similar industries may both be subject to the same selection in and out of occupation and may, furthermore, be rather similar in respect to education and income, thereby limiting the confounding effects of social factors.

Gilbert recommended that a lag of at least one or two years would seem essential in any analysis and for certain chronic nonmalignant diseases longer lags may be necessary [24], which is in line with our results. The rationale seemed to be that since only the healthier workers survived on the job to cumulate recent exposures, those recent exposures should be ignored. For a lag to be successful in minimizing HWE bias, the period that HWE operates must be shorter than the lag period [24]. It should be emphasized that our suggested lag time of three or more years may only be relevant for our specific outcome (all-cause mortality) [15].

Contrary, restricting the analysis to persons actively employed [12,14,24] did not minimize HWE bias in that several MMRs were further away from unity compared to the model without restriction. This may be consistent with Arrighi [12,14], who argued that employment status may be an intermediate variable between exposure and outcome and restriction could introduce collider bias between Elec0 and UH1, resulting in a negative association between Elec0 and UH1. Elec0 would therefore have a detrimental effect on Mort3 (Figure 1). This was supported in our data by the increased risk for electricians only in year 0 when comparing Model 2 with Model 3 for all reference groups (Tables 4, 5 and 6). To account for this collider bias we implemented the marginal structural model, which rather surprisingly did not minimize HWE bias (Tables 4, 5 and 6 - Model 4). One reason could be that employment status in year 1 (Emp1) was only partly associated with UH1 and that the setup in Figure 1 therefore was incorrect. We conclude that MSM did not appear to minimize HWE bias in our study.

Other authors suggest restriction to newly employed workers [5,9], which seemed supported in our study, but was based on a low number of cases. Analysis of long-term employed electricians showed almost the same result as the general result (Table 4) and should not be recommended.

From a methodological standpoint, selection bias and confounding are two concepts that often overlap [29], which is also seen in the literature concerning HWE. Some authors argue that HWE is selection bias, which occurs because persons in poor health are selected out of the workforce [5,11,12,15,30]. Other authors state that HWE is due to the selection of an inappropriate reference group, typically the general population, which contains the chronically ill and hospitalized and person otherwise unfit to seek and maintain employment [3,12]. Other authors suggest that incomplete follow-up of the section of workers who leave employment could be a source of HWE [5,31-33], while other again consider HWE as confounding in that the (unmeasured) health status of the group of employees affects the mortality outcome [5,12,34]. Rothman argues that although HWE has traditionally been classified as selection bias, HWE is the influence of another factor that influences both worker status and health and, as such, HWE is an example of confounding [29]. Checkoway states that HWE is perhaps the most common example of confounding in occupational studies [3]. We agree with the last viewpoint that in case of complete follow-up, HWE is an example of confounding by health status that predicts employment as well as mortality outcome.

Our study has several strengths, e.g. we have information on all Danish men aged 26-56 years at baseline and we have complete follow-up until death or emigration of all Danish citizens. The results of the study are therefore not influenced by missing outcome information on those individuals who leave the work force before retirement [5,31-33] or different outcome information between electricians and the reference groups [12], which are great qualities of research in Danish and other Nordic countries [35]. In countries without such data, appropriate reference group selection may not be possible, but our result of minimizing of HWE bias by lag time analyses may be applicable. Since all Danish men are included, representativeness of the results was ensured [36]. Furthermore, data sources were comparable due to the fact that information on social events, morbidity and mortality was obtained from the same data sources on the whole population [12]. Furthermore, we included information on public income substitutions provided by the Danish welfare state, which is the main supplier of income compensations in Denmark. The collection of the register data was collected independently of the research project, which leaves less room for recall and non-response bias [36]. Finally, it is a strength of the study that we focus on a short time span (only three years) since we are able to show the influence of employment during only few years.

We also wish to emphasize the limitations of our study. We used administrative registers, which primarily contains information relevant to public sector administrators, e.g. when using information on sick leave, we only had information on the number of days of sick leave compensation and e.g. not the reasons for the compensation [36]. Another limitation was that only information on a few confounding factors was available; other unmeasured confounders could have been added to Figure 1. Finally, misclassification of administrative data, especially misclassification of information on specific employment based on occupational position code, highest vocational education and line of business, may be present, but we deem this non-differential misclassification since it was unrelated with mortality years later. Non-differential misclassification tends to underestimate the association between employment and mortality risk, which means that the magnitude of HWE may have been underestimated in our study. We also have to underscore that HWE is used as the only explanation for the lower mortality among employed electricians compared to the general population. If information were available, this association could be divided into the effects of underlying health, lifestyle or socioeconomic factors or other factors explaining the differences in mortality.

## Conclusions

We observed a strong selection out of the cohort with increased mortality among electricians leaving the work force. The odds ratio of re-entering was low for persons on long-term sick leave, unemployment periods, incapacity benefits and with increased comorbidity index. The HWE was strong with 40% decreased mortality among active employed electricians compared to the general population; adjustment for several social variables and comorbidity index slightly attenuated this result. When including relevant reference groups and inferring lag time these differences were minimized. We conclude that HWE is a strong selection process in occupational cohorts and that the bias that arises due to this phenomenon can be minimized by appropriate reference selection and lag time analyses.

## List of abbreviations

95%CI: 95% confidence intervals; Emp1: employment in any occupation in year 1; Elec0: employment as electrician in year 0; Elec2: employment as electrician in year 2; HWE: healthy worker effect; HWE bias: bias caused by the healthy worker effect; Mort3: mortality in year 3; MR: mortality rate; MRR: mortality rate ratios; MSM: marginal structural model; OR: odds ratio; UH1: underlying health in year 1.

## Competing interests

The authors decleare that they have no competing interests.

## Authors' contributions

LCT designed the study and participated in analysis and interpretation of data, drafting of the manuscript, had full access to all of the data in the study and performed the statistical analysis. UAH participated in developing the study concept and design, in the analysis and interpretation of data and helped with critical revision of the manuscript for important intellectual content. SM and HBH both participated in the interpretation of data and helped with critical revision of the manuscript for important intellectual content. All authors read and approved the final manuscript.

## Pre-publication history

The pre-publication history for this paper can be accessed here:

http://www.biomedcentral.com/1471-2458/11/571/prepub
